# Hero or sidekick? Organellar reactive oxygen species during abscisic acid-induced stomatal closure

**DOI:** 10.1093/plphys/kiad080

**Published:** 2023-02-10

**Authors:** Divya Mishra

**Affiliations:** Assistant Features Editor, Plant Physiology, American Society of Plant Biologists, USA; Department of Botany, University of Wisconsin, Madison 53706, USA

The phytohormone abscisic acid (ABA) is essential during plant responses to various abiotic stresses, including drought. ABA increases under drought stress, eventually leading to stomatal closure and simultaneously reducing photosynthesis (Kalladan et al. 2017). Guard cells closely regulate stomatal aperture to maintain efficient photosynthesis with minimal water loss (Jezek and Blatt 2017).

The ABA signaling cascade possesses three constituents: ABA receptor, group-A protein phosphatase type 2C (PP2C), and sucrose non-fermenting 1 (SNF1)-related protein kinase 2 (SnRK2). In the presence of ABA, the ABA receptor perceives and inhibits PP2C, and phosphorylation of SnRK2 increases and activates the downstream processes (Weiner et al. 2010; Ruschhaupt et al. 2019). Respiratory burst oxidase homologs (RBOH) are the downstream targets of SnRK2 and are activated through phosphorylation. RBOH is a multigene family and a major source of reactive oxygen species (ROS). RBOH-mediated ROS production in the presence of ABA triggers signaling to close the stomata (Jezek and Blatt 2017; Watkins et al. 2017). While many aspects of ROS signaling are now well-established, still many questions remain to be explored.

In a recent issue of *Plant Physiology*, Postiglione and Muday (2023) studied the intracellular origin sites of ABA-induced ROS during stomatal closure in Arabidopsis (*Arabidopsis thaliana*) with the help of robust ROS detection methodology. Using the generic ROS-responsive fluorescent probe CM 2,7-dihydro-dichlorofluorescein diacetate (CM H2DCF-DA), the authors observed that ABA enhances ROS production in distinct subcellular regions, including the mitochondria, chloroplasts, nucleus, and cytosol. The authors found no change in DCF fluorescence in the mitochondria, cytosol, and nucleus of an ABA receptor mutant, which suggested that ABA increases ROS levels in different subcellular compartments. To understand how ROS, particularly H_2_O_2,_ increase in response to ABA, the authors used the H_2_O_2_-specific probe Peroxy Orange 1 (PO1). An increase in PO1 signal was observed only in chloroplasts but not in the mitochondria, nucleus, or cytosol at low concentrations of ABA. Under higher concentrations of ABA, they observed significant PO1 signals from the mitochondria and chloroplasts, demonstrating that chloroplasts and mitochondria are the sites of ABA-induced H_2_O_2_ production.

There are certain limitations in using fluorescent dye and chemical probes to visualize changes in ROS, including irreversibility and differential dye uptake by some organelles (Martin et al. 2022). Therefore, the authors also used the genetically encoded ROS biosensor roGFP2-Orp1 that provides the oxidation ratio. ABA treatment resulted in increased oxidation ratios of whole stomata, nucleus, and cytosol in roGFP2-Orp1 lines. Further, the authors used the mitochondrial-specific biosensor (mt-roGFP2-Orp1) for visualizing H_2_O_2_ accumulation in guard cell mitochondria upon ABA treatment. The oxidation of mt-roGFP2-Orp1 increased in the presence of ABA but not uniformly or intensely like the cytosolic chemical ROS probe. The results obtained with biosensors and chemical sensors indicate similar phenomena of mitochondrial ROS accumulation upon ABA treatment during stomatal closure.

**Figure 1.**
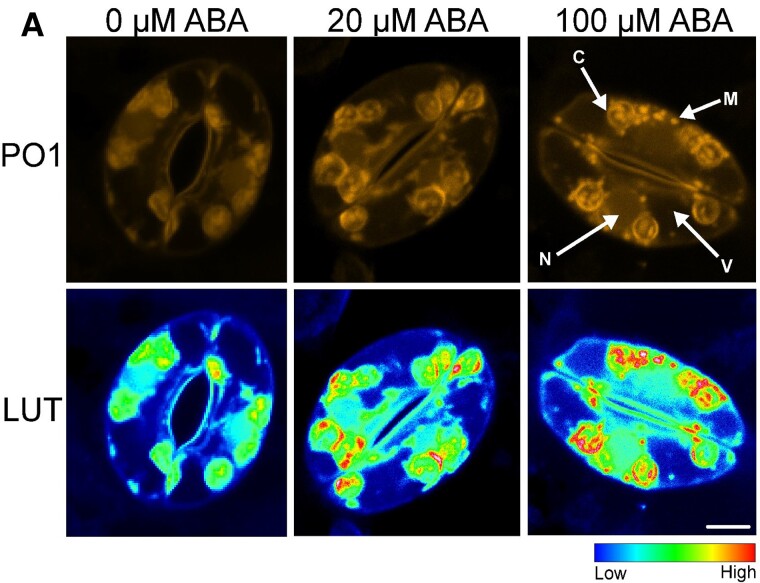
ABA increases organellar H_2_O_2_ during stomatal closure. A. Confocal micrographs of Arabidopsis guard cells stained with H_2_O_2_-specific chemical probe peroxy orange 1 (PO1) under different concentrations of ABA. Scale bar: 5 µm. The figures are adapted from Postiglione and Muday (2023). LUT, lookup tables; M, mitochondria; V, vacoule; N, nucleus; ABA, abscisic acid.

To further address the role of ABA-induced ROS in the mitochondria of guard cells, the authors used the *ABA overly sensitive 6* (*abo6*) mutant that shows an enhanced response to ABA. They pretreated *abo6* mutants with a mitochondrial-specific ROS enhancer and observed rapid stomatal closure under ABA treatment. In contrast, pretreatment with mitochondrially targeted antioxidant suppressed stomatal closure and weakened the hypersensitive ABA-responsive nature of *abo6*. Further, they observed no fluorescence in *rbohD rbohF* double mutant mitochondria under ABA application. Collectively, these results demonstrate that mitochondrial ROS contribute to ABA-induced stomatal closure (Fig. 1). This detailed study on sources and factors contributing to organellar ROS production provides insight into whether organellar ROS are essential or a “byproduct” of general signaling events during stomatal closure.
